# Effects of metformin on tumor hypoxia and radiotherapy efficacy: a [^18^F]HX4 PET imaging study in colorectal cancer xenografts

**DOI:** 10.1186/s13550-019-0543-4

**Published:** 2019-08-02

**Authors:** Sven De Bruycker, Christel Vangestel, Steven Staelens, Leonie wyffels, Jan Detrez, Marlies Verschuuren, Winnok H. De Vos, Patrick Pauwels, Tim Van den Wyngaert, Sigrid Stroobants

**Affiliations:** 1 Molecular Imaging Center Antwerp (MICA), University of Antwerp, Universiteitsplein 1, Antwerp, 2610 Belgium; 2 Department of Nuclear Medicine, Antwerp University Hospital (UZA), Wilrijkstraat 10, Edegem, 2650 Belgium; 3 Laboratory of Cell Biology and Histology, University of Antwerp, Universiteitsplein 1, Antwerp, 2610 Belgium; 4 Center for Oncological Research (CORE), University of Antwerp, Universiteitsplein 1, Antwerp, 2610 Belgium; 5 Department of Pathology, Antwerp University Hospital (UZA), Wilrijkstraat 10, Edegem, 2650 Belgium

**Keywords:** [^18^F]HX4, PET, CT, Hypoxia, Imaging biomarker, Metformin, Radiotherapy, Cancer

## Abstract

**Background:**

In a colorectal cancer xenograft model, we investigated the therapeutic effect of metformin on tumor hypoxia with [^18^F]flortanidazole ([^18^F]HX4) small-animal positron emission tomography (μPET). We also assessed the additive effect of metformin on long-term radiotherapy outcome and we studied the potential of [^18^F]HX4 as a predictive and/or prognostic biomarker within this setup.

**Methods:**

Colo205-bearing mice (*n *= 40) underwent a baseline [^18^F]HX4 hypoxia μPET/computed tomography (CT) scan. The next day, mice received 100 mg/kg metformin or saline intravenously (*n *= 20/group) and [^18^F]HX4 was administered intravenously 30 min later, whereupon a second μPET/CT scan was performed to assess changes in tumor hypoxia. Two days later, mice were further divided into four therapy groups (*n *= 10/group): control (1), metformin (2), radiotherapy (3), and metformin + radiotherapy, i.e., combination (4). Then, they received a second dose of metformin (groups 2 and 4) or saline (groups 1 and 3), followed by a single radiotherapy dose of 15 Gy (groups 3 and 4) or sham irradiation (groups 1 and 2) 30 min later. Tumor growth was followed three times a week by caliper measurements to assess the therapeutic outcome.

**Results:**

[^18^F]HX4 uptake decreased in metformin-treated tumors with a mean intratumoral reduction in [^18^F]HX4 tumor-to-background ratio (TBR) from 2.53 ± 0.30 to 2.28 ± 0.26 (*p* = 0.04), as opposed to saline treatment (2.56 ± 0.39 to 3.08 ± 0.39; *p* = 0.2). The median tumor doubling time (TDT) was 6, 8, 41, and 43 days in the control, metformin, radiotherapy and combination group, respectively (log-rank *p* < 0.0001), but no metformin-specific therapy effects could be detected. Baseline [^18^F]HX4 TBR was a negative prognostic biomarker for TDT (hazard ratio, 2.39; *p* = 0.02).

**Conclusions:**

Metformin decreased [^18^F]HX4 uptake of Colo205-tumors, but had no additive effect on radiotherapy efficacy. Nevertheless, [^18^F]HX4 holds promise as a prognostic imaging biomarker.

## Background

Metformin, an antidiabetic that reduces hepatic gluconeogenesis, has gained significant interest in the oncological field over the last decades. This interest in metformin for cancer treatment stems from clinical observations in diabetes patients that metformin use was associated with significantly lower cancer incidence and improved prognosis [1]. Since then, the use of metformin as an anticancer therapeutic in non-diabetic cancer patients, including colorectal cancer (CRC) [2, 3], has been tested in different clinical trials, from which the first results are encouraging [2, 4]. Studies aiming at elucidating metformin’s anti-cancer properties have revealed a complex interplay with different molecular targets, including adenosine monophosphate-activated protein kinase (AMPK), mammalian target of rapamycin (mTOR) complex 1 and complex I of the mitochondrial electron transport chain (ETC) [5]. Moreover, by reducing insulin/insulin-like growth factor-1 signaling, metformin may also systemically inhibit cancer development [4].

Metformin’s direct inhibitory effects on complex I of the ETC may at least partially explain the potentiated radiotherapy response observed in diabetes patients with cancer who underwent radiotherapy [5–7] and in different non-diabetic xenograft models treated with metformin and irradiation [8–10]. Indeed, inhibition of the ETC results in a decrease in cellular oxygen consumption and thus reoxygenation of hypoxic cells [8]. This is crucial for radiotherapy to be effective, since the oxygen radicals (as formed by radiation) may inflict damage that is more difficult to repair, as postulated in the ‘oxygen fixation’ hypothesis [11].

To fully exploit the results of current and future prospective clinical trials in which metformin is being investigated as a radiosensitizer, biomarkers are crucial. Non-invasive molecular imaging such as positron emission tomography (PET) offers advantages over other techniques [12]. For instance, imaging biomarkers allow non-invasive and serial studies of the entire tumor mass in vivo, thereby coping with intratumoral heterogeneity. They can confirm the specificity of on-target drug effects, provide evidence of biological activity and identify patients who are the most likely to benefit. Moreover, PET can help to evaluate drug efficacy much earlier than anatomical imaging techniques [12, 13].

Seeing the hypoxia-modulating effects of metformin, quantification of tumor hypoxia with PET may be very useful as a biomarker. Many of the developed radiotracers that accumulate in hypoxic tissue are ^18^F-labeled 2-nitroimidazole derivatives, such as [^18^F]flortanidazole ([^18^F]HX4). Only under conditions of low oxygenation (i.e., ≤ 10 mmHg), these molecules are reduced and the resulting reactive intermediates are retained by viable hypoxic cells, but not by apoptotic or necrotic cells. In the presence of oxygen, the intermediate products are re-oxidized into the parent 2-nitroimidazole compound which diffuses out of the cell [14].

In a previous study, we demonstrated that [^18^F]HX4 PET holds potential as a predictive and prognostic imaging biomarker in an A549 non-small cell lung cancer (NSCLC) xenograft model treated with metformin and single-dose radiotherapy [10]. Our results support performing further clinical trials to assess the value of baseline [^18^F]HX4 PET to identify NSCLC patients who could potentially benefit from adding metformin to radiotherapy, since metformin’s therapeutic benefit may depend on the baseline degree of tumor hypoxia. However, also tumor-specific differences may influence the ability of hypoxic cell-radiosensitizing therapeutics such as metformin [5]. At present, different phase II clinical trials have been set up in which metformin is incorporated in chemoradiotherapy in non-diabetic CRC patients (e.g., NCT02437656, NCT03053544). Therefore, we investigate herein the potential of [^18^F]HX4 PET in the Colo205 CRC xenograft model in which we have previously shown substantial degrees of baseline hypoxia [15, 16]. In order to gain a better understanding of the underlying mechanisms of our in vivo observations, we performed an ex vivo validation experiment in a separate cohort of tumor-bearing mice.

## Methods

### Animal model

The experimental protocol was approved by the Antwerp University Ethical Committee for Animal Experiments (2018-04) and all experiments were performed in accordance with European and Belgian regulation. Female CD-1 athymic nude mice at an age of 6–7 weeks (*n* = 64; Charles River) were group-housed (up to six animals per cage) in individually ventilated cages under a 12-h light/dark cycle in a temperature- and humidity-controlled environment, provided with certified rodent diet and fresh water ad libitum, and cage enrichment.

Human Colo205 CRC cells (PerkinElmer) were routinely cultured in RPMI 1640 medium (Invitrogen) as previously described [15]. Colo205 cells were harvested by trypsinization with 0.05% trypsin-EDTA (Invitrogen), washed two times with sterile phosphate-buffered saline, counted using the Muse Cell Count and Viability Assay (Merck Millipore) and resuspended in sterile phosphate-buffered saline at a concentration of 2 × 10^7^ viable cells per milliliter. Mice were inoculated with 100 μL cell suspension in the right hind leg (*n* = 40; imaging study) or both hind legs (*n* = 24; histology study). When tumors became palpable, tumor diameters were measured with a digital caliper three times a week and tumor volumes were approximated with the formula 0.5 × length × width^2^.

### Radiotracer production

[^18^F]HX4 was prepared as previously described [10]. Radiotracer was obtained with a radiochemical purity of more than 99% and a radiochemical yield of 23% ± 3% (decay-corrected to end of bombardment; *n* = 8). The molar activity was 346 ± 51 GBq/μmol (decay-corrected to end of synthesis; *n* = 8).

### Experimental setup

#### Imaging study

**Fig. 1:**
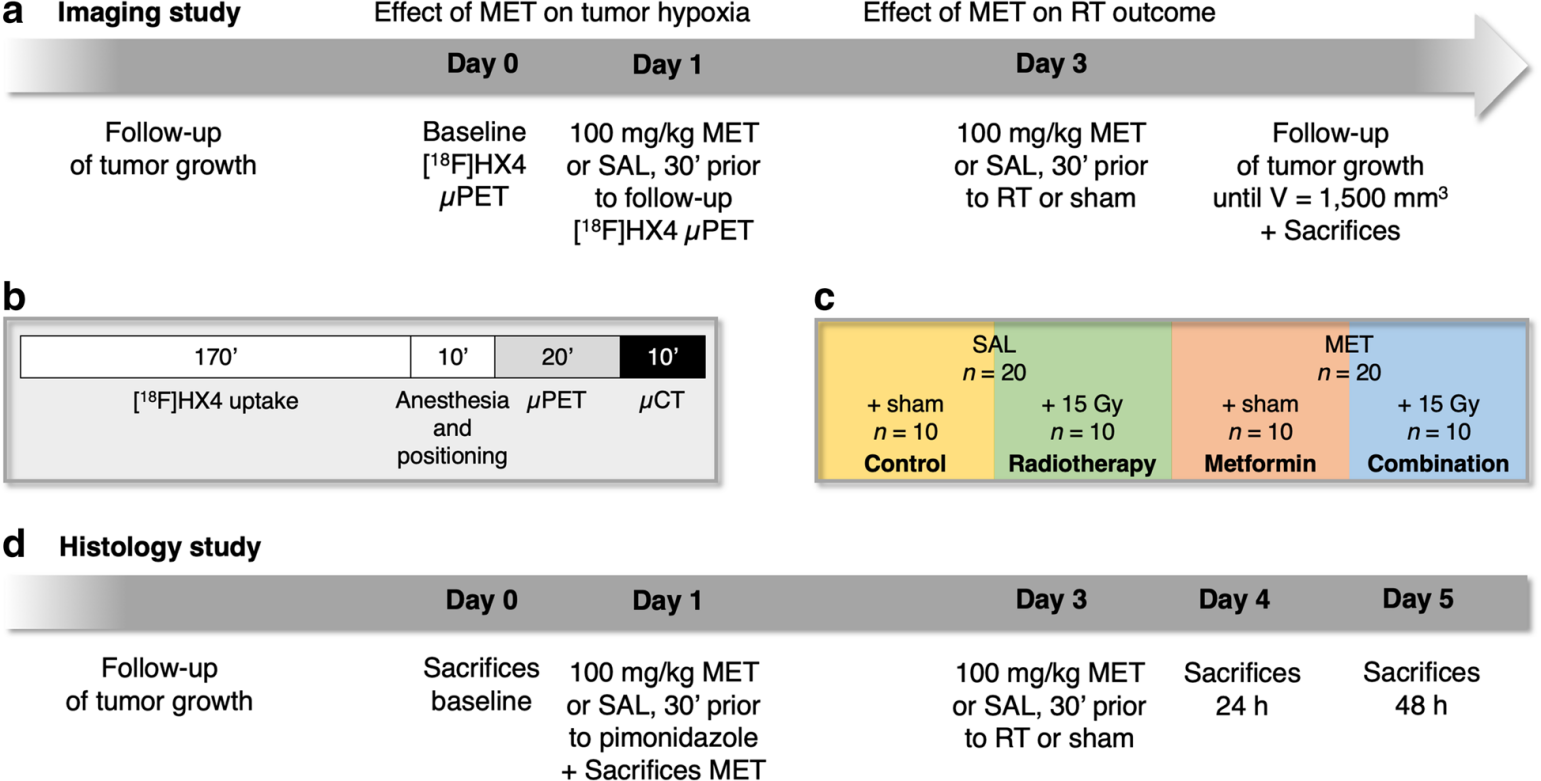
Study design. **a** Imaging study. Animals underwent a baseline [^18^F]HX4 small-animal PET (μPET) scan and were rescanned 30 min after metformin or saline administration 24 h later (i.e., follow-up scan). Two days later, animals were given therapy or sham and tumor volume was followed until a volume of 1500 mm^3^ was reached. **b** Detail of the [^18^F]HX4 μPET/CT scan protocol. **c** Overview of the different therapy groups. **d** Histology study. Animals were sacrificed at different points in time for ex vivo validations of baseline and early post-therapy parameters. MET, metformin; RT, radiotherapy; SAL, saline

The study design is shown in Fig. 1a. Colo205 tumor-bearing mice with a mean tumor volume of 232 ± 22 mm^3^ mice underwent a baseline [^18^F]HX4 scan (=day 0). Approximately 18.5 MBq in a final volume of 200 μL saline was administered as a bolus injection via the tail vein. During image acquisition, which started 180 min after tracer administration [17], the animals were anesthetized with isoflurane (induction, 5%; maintenance, 1%–2%; Abbott) and medical oxygen (100%), body temperature was kept constant at 37°C using a feedback-controlled warm airflow (Minerve), and respiration was continuously monitored using the Monitoring Acquisition Module (Minerve). A static small-animal PET (μPET) acquisition (20 min) followed by an 80 kV/500 μA computed tomography (CT) acquisition (10 min) was performed on an Inveon μPET/CT scanner (Siemens Preclinical Solutions). Figure 1b summarizes the scan protocol. The next day (=day 1), mice were divided into two groups to achieve comparable tumor volumes (333 ± 50 mm^3^ vs. 286 ± 43 mm^3^, respectively; *p* = 0.4) and were given a 100 mg/kg-dose of metformin hydrochloride (ABC Chemicals) in a final volume of 100 μL of saline (*n* = 20) or the same volume of saline alone (*n* = 20) intravenously. Thirty minutes later, the animals were injected with approximately 18.5 MBq of [^18^F]HX4 intravenously, whereupon a follow-up μPET/CT scan was performed to assess changes in tumor hypoxia (Fig. 1a). One mouse could not be rescanned due to unsuccessful tracer administration.

PET images were reconstructed using 4 iterations × 16 subsets of a 3-dimensional ordered subset expectation-maximization algorithm after Fourier rebinning. Normalization, as well as correction for dead time, scatter, and attenuation, was applied. The μPET/CT images were analyzed with PMOD v3.3 software (PMOD Technologies). An elliptic volume-of-interest that enclosed the entire tumor was positioned manually and was centered on the tumor area that showed maximal radiotracer uptake. Then 3-dimensional isocontours at 60% of the maximum pixel value within this volume-of-interest were drawn automatically. TBRs were determined using heart, dominated by the blood pool contributions, as a measure of vascular background. The heart was manually delineated on the CT images of each mouse [10]. Tumors were also manually delineated on the CT images for determination of the tumor volumes.

Two days after the follow-up scan (=day 3), tumor-bearing mice that received metformin were further divided into a metformin monotherapy group and a metformin + radiotherapy group (hereinafter referred to as “combination”; *n* = 10/group). Mice that received saline were further divided into a control group and a radiotherapy group (*n* = 10/group; Fig. 1c). All groups had comparable body weight, tumor volume and baseline [^18^F]HX4 uptake (Table 1). The animals of the metformin groups received a second 100 mg/kg-dose of metformin hydrochloride, and the mice of the saline groups were administered saline. Thirty minutes after treatment, a single dose of radiotherapy was administered to the radiotherapy groups. The control group and the metformin monotherapy group received sham irradiation. In short, during irradiation, the animals were anesthetized with isoflurane (induction, 5%; maintenance, 1%–2%) and positioned within the self-contained X-ray system XRAD 320 (Precision X-Ray). The whole body of the animals was shielded using lead, except for the tumor-bearing leg. Irradiation was delivered at a rate of 100 cGy/min with 320 kV X-rays. Tumors received a single dose of 15 Gy. Animals that received sham irradiation were anesthetized and positioned within the X-ray system for 15 min but were not irradiated. After the irradiation experiment, growth of tumors was monitored until they reached a volume of 1500 mm^3^ (the ethical endpoint of the study). After reaching the endpoint, animals were sacrificed and tumors were collected for ex vivo validations on tumor tissue. The tumor doubling time (TDT), that is, the time to reach twice the baseline volume, was used as a proxy for progression-free survival (i.e., the proportion of animals without tumor doubling) and was defined as the endpoint.

**Table 1:** Overview of the baseline parameters of the four treatment groups of the imaging study

	Control	Metformin	Radiotherapy	Combination	*P* value
Animal weight (g)	24.7 ± 0.6	24.2 ± 0.9	24.7 ± 0.6	24.2 ± 0.5	1.0
Tumor volume (mm^3^)	382 ± 68	436 ± 79	336 ± 80	389 ± 81	0.7
Baseline [^18^F]HX4 TBR	2.33 ± 0.34	2.48 ± 0.41	2.78 ± 0.71	2.59 ± 0.46	1.0

Data are expressed as mean ± SEM. TBR, tumor-to-background ratio

#### Histology study

To investigate the immediate therapy effects of metformin and radiotherapy on tumor hypoxia, proliferation and apoptosis rate, a satellite study was performed on a separate cohort without imaging for the purpose of histological analysis. The study design is shown in Fig. 1d. Two weeks after inoculation, Colo205 tumors reached a volume of 254 ± 24 mm^3^. At this point, 1 h after intraperitoneal administration of 60 mg/kg pimonidazole hydrochloride (Hypoxyprobe), four animals were euthanized via cervical dislocation for the ex vivo evaluation of the baseline tumor parameters. One day later, four animals received a single 100 mg/kg dose of metformin hydrochloride intravenously, and 60 mg/kg pimonidazole hydrochloride intraperitoneally 30 min later. After 1 h, animals were euthanized. The next day, the remaining animals (*n* = 16) were divided into four therapy groups with comparable body weight and tumor volume (*n* = 4/group; Table 2) which underwent the same treatments as their counterparts of the imaging study (Fig. 1c). Twenty-four hours and 48 h later, respectively, 2 randomly chosen animals per group were administered pimonidazole and euthanized 1 h later, for the ex vivo evaluation of early therapy effects.

**Table 2:** Overview of the baseline parameters of the four treatment groups of the histology study

	Control	Metformin	Radiotherapy	Combination	*P* value
Animal weight (g)	28.3 ± 1.6	26.5 ± 0.9	26.3 ± 0.6	26.1 ± 1.3	0.7
Tumor volume (mm^3^)	307 ± 73	371 ± 72	390 ± 99	402 ± 81	0.8

Data are expressed as mean ± SEM

### Immunohistochemistry

Immediately following sacrifice of the animals, tumor tissue was resected, formalin-fixed and paraffin-embedded. Tissue sections of 3 mm thick were mounted on SuperFrost microscope slides (Menzel-Glaser) for immunostaining with primary antibodies targeting Ki67 (1:300; Cell Signaling Technology, #9027), cleaved caspase-3 (CC3; 1:300; Cell Signaling Technology, #9661) and a pre-conjugated pimonidazole-fluorescein isothiocyanate (FITC; 1:100; Hypoxyprobe; not for the animals of the imaging study); and secondary anti-rabbit horseradish peroxidase (HRP)-labeled SignalStain Boost detection reagent (ready-to-use; Cell Signaling Technology; for Ki67 and CC3) or anti-FITC antibody labeled with HRP (1:100; Hypoxyprobe; for pimonidazole). Stainings were performed according to the manufacturers’ instructions.

Slices were examined under a CX31 light microscope (Olympus). Ki67 staining was scored by two independent researchers. For quantification of CC3 and pimonidazole staining, 5–15 fields of 1 section per tumor were photographed at × 200 magnification (0.17 μm/pixel) using a DS-Fi1 camera (Nikon) and specialized NIS-Elements D software (Nikon), thereby avoiding areas of high necrosis. From these pictures, the mean total percentages of CC3 and pimonidazole, respectively, were semi-automatically quantified using a customized segmentation script (available upon request) based on spectral deconvolution and channel-dependent thresholding, implemented in Fiji software (ImageJ).

### Statistics

Changes in [^18^F]HX4 TBR were evaluated with a Wilcoxon matched-pairs signed-rank test. In vivo baseline parameters and ex vivo immunohistochemistry data were analyzed using Mann-Whitney *U* tests, or Kruskal-Wallis tests with Dunnett’s post-hoc analysis (Prism 8, GraphPad Software). Non-parametric tests were used since most of the in vivo data were not normally distributed and the ex vivo sample size was limited. Data are expressed as mean ± standard error of the mean (SEM).

Differences in TDT were analyzed using the Kaplan-Meier technique (with log-rank test) and Cox proportional hazards regression (Stata 15.1, StataCorp LLC). Therapy-specific effects were assessed by estimating the interaction term of treatment and [^18^F]HX4 uptake. [^18^F]HX4 TBR (which was not normally distributed) was log-transformed for these analyses. Model checks for goodness-of-fit and proportional hazards assumption were performed as appropriate. Results are reported as hazard ratios (HR) with 95% confidence intervals (CI). *P* values < 0.05 were considered statistically significant.

## Results

### Imaging study

#### Metformin reduces tumor hypoxia in Colo205 tumors

**Fig. 2:**
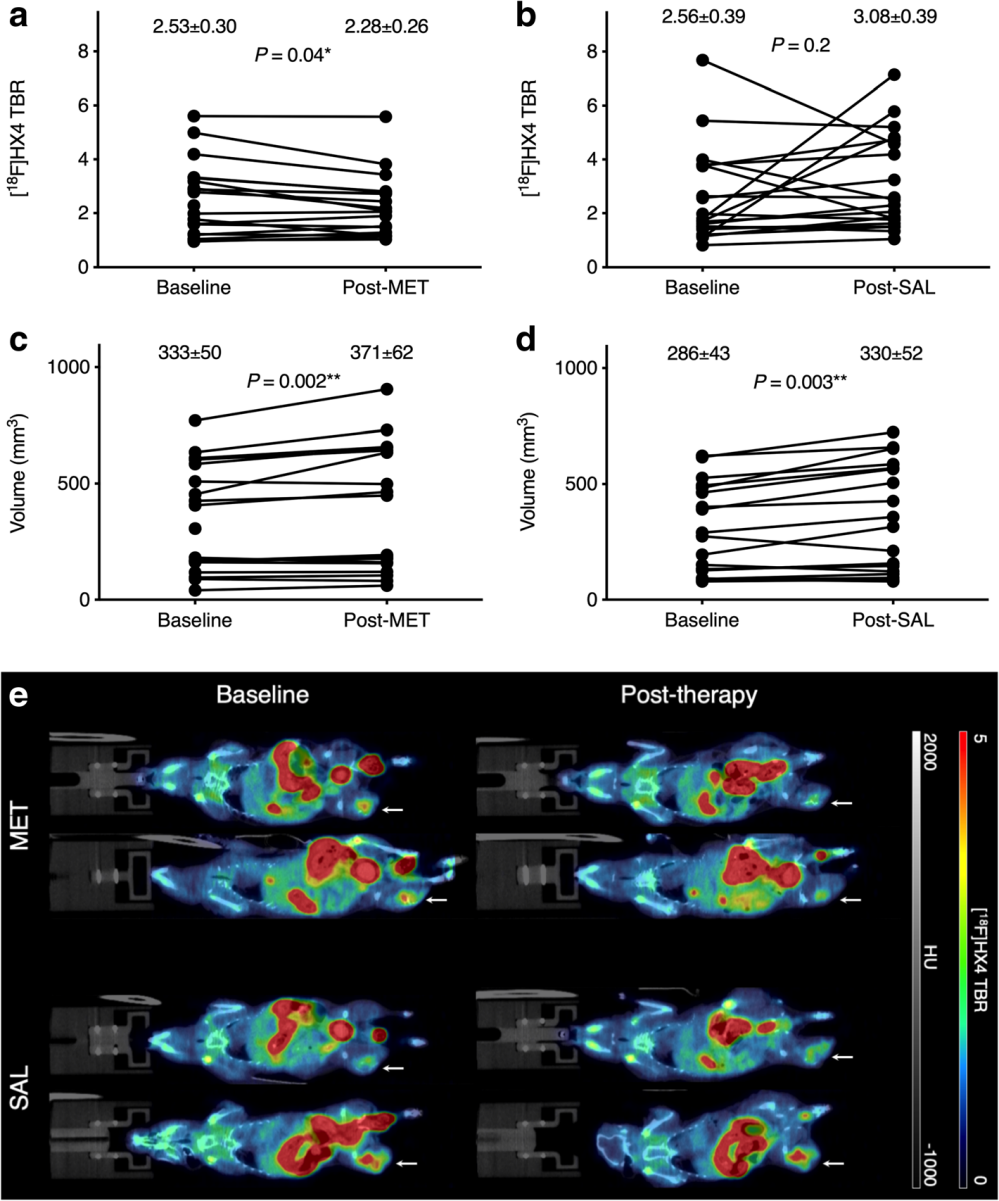
[^18^F]HX4 small-animal PET/CT (μPET/CT) imaging results. **a** [^18^F]HX4 tumor-to-background ratio (TBR) decreased after metformin (MET) administration (*p* = 0.04). **b** Saline (SAL) administration had no influence on tumor hypoxia as measured with [^18^F]HX4. **c** Tumor volumes in the metformin cohort significantly increased between the baseline scan and the follow-up scan 24 h later (*p* = 0.002). **d** The same significant increase in tumor volume was measured in the saline cohort (*p* = 0.003). **e** Representative [^18^F]HX4 TBR-corrected μPET/CT images of 2 metformin-treated and 2 saline-treated animals before and after metformin administration (coronal view). Arrows indicate tumors. HU, Hounsfield unit

In animals treated with metformin 30 min prior to tracer injection, [^18^F]HX4 TBR decreased from 2.53 ± 0.30 at baseline to 2.28 ± 0.26 (*p* = 0.04; Fig. 2a), but not in the animals treated with saline (2.56 ± 0.39 to 3.08 ± 0.39; *p* = 0.2; Fig. 2b). The mean tumor volume in both metformin- and saline-treated mice increased between the baseline and the follow-up scan 1 day later (333 ± 50 mm^3^ to 371 ± 62 mm^3^; *p* = 0.002 (Fig. 2c) and 286 ± 43 mm^3^ to 330 ± 52 mm^3^; *p* = 0.003 (Fig. 2d), respectively). Figure 2e shows representative [^18^F]HX4 PET/CT images of metformin- and saline-treated animals before and after therapy administration.

#### Metformin does not increase radiosensitivity in Colo205 tumors

**Fig. 3:**
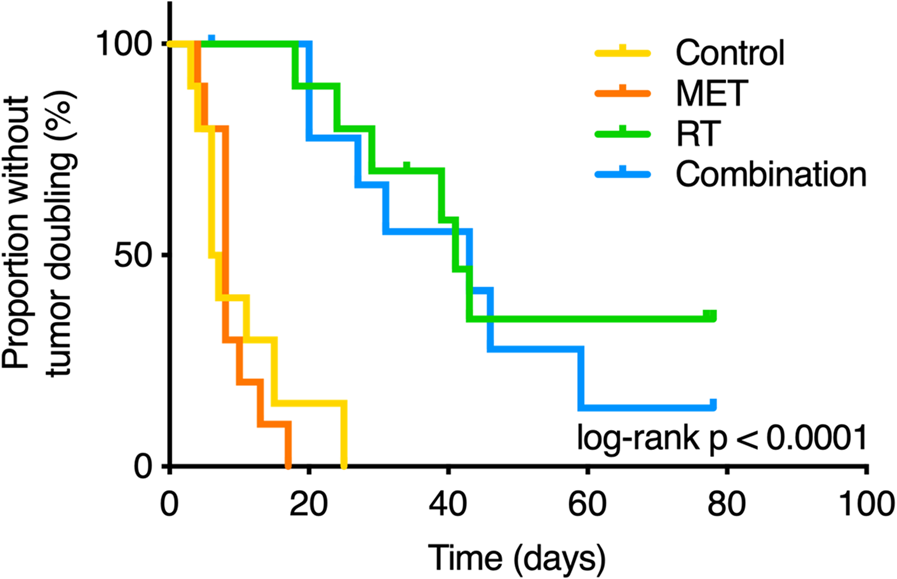
Kaplan-Meier representation of the proportion of animals without tumor doubling based on therapy regimen (overall log-rank *p* < 0.0001). MET, metformin; RT, radiotherapy

Figure 3 shows how a single dose of metformin did not exert a significant effect on TDT (median TDT 8 days vs. 6 days compared to controls; HR, 1.36; 95% CI, 0.51–3.65; *p* = 0.5). In comparison, radiotherapy significantly extended median TDT to 41 days (HR, 0.05; 95% CI, 0.01–0.18; *p* < 0.0001) compared to controls. Metformin had however no additive effect on the radiotherapy response in this model since median TDT only increased with 2 days compared to radiotherapy alone (median TDT 43 days; HR, 1.31; 95% CI, 0.44–3.91; *p* = 0.6). Moreover, a complete tumor eradication was observed in 3 of the 10 animals after radiotherapy, but not in any of the other groups.

**Fig. 4:**
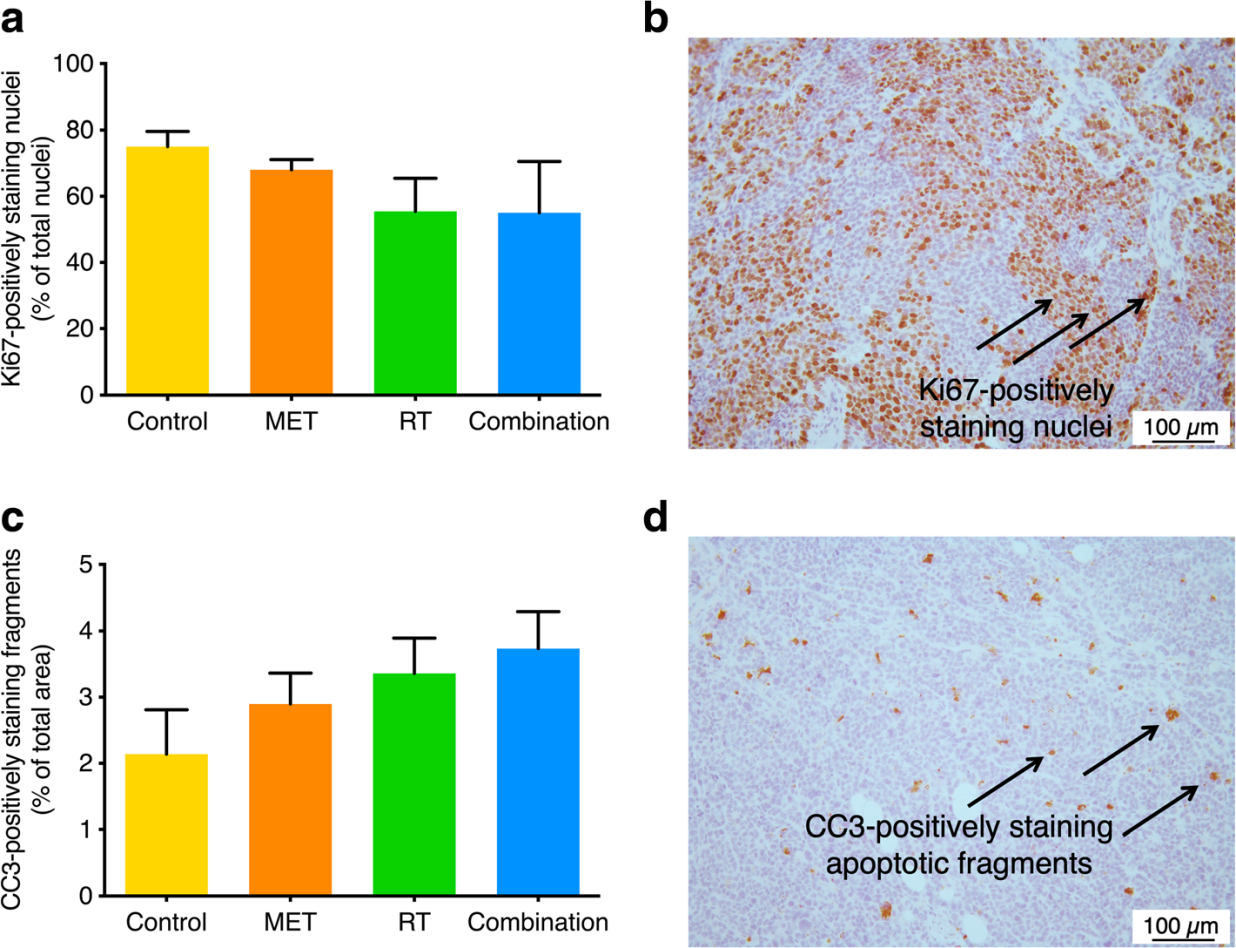
Ex vivo parameters at time-of-sacrifice (i.e., a tumor volume of 1500 mm^3^). **a** Proliferation as assessed by the percentage of Ki67-positive nuclei. **b** Representative Ki67-stained tumor tissue of a metformin (MET)-treated animal. Arrows indicate Ki67-positively staining nuclei. **c** Apoptosis as assessed by the percentage of cleaved caspase-3 (CC3)-positive fragments. **d** Representative CC3-stained tumor tissue of a metformin-treated animal. Arrows indicate CC3-positively staining apoptotic fragments. Statistical significance was not reached. RT, radiotherapy

At time-of-sacrifice (i.e., a tumor volume of 1500 mm^3^), tumor cell proliferation (as measured by the relative number of Ki67-positive nuclei in the tumor) was visually, but not significantly decreased in radiotherapy-treated (55% ± 10%) and combination-treated (55% ± 15%) animals compared to controls (75% ± 5%) and metformin-treated (68% ± 3%) animals (*p* = 0.4; Fig. 4a, b). Accordingly, visually, but not significantly more apoptosis (determined by the relative number of CC3-positive cells in tumor) was detected in radiotherapy-treated (3.36% ± 0.53%) and combination-treated (3.74% ± 0.56%) tumors compared to controls (2.14% ± 0.62%) and metformin-treated (2.47% ± 0.23%) animals (*p* = 0.2; Fig. 4c, d).

#### [^18^F]HX4 PET shows promise as a prognostic biomarker

Controlling for the effect of radiotherapy showed that a 10.0% increase in [^18^F]HX4 TBR was associated with an 8.6% increase in the hazard of tumor doubling (*p* = 0.02). Thus, baseline [^18^F]HX4 PET holds potential as a prognostic biomarker for TDT within this setup. However, neither baseline [^18^F]HX4 TBR nor the change in [^18^F]HX4 TBR at follow-up had a predictive value for radiotherapy treatment effect in the Colo205 tumor model.

### Histology study

**Fig. 5:**
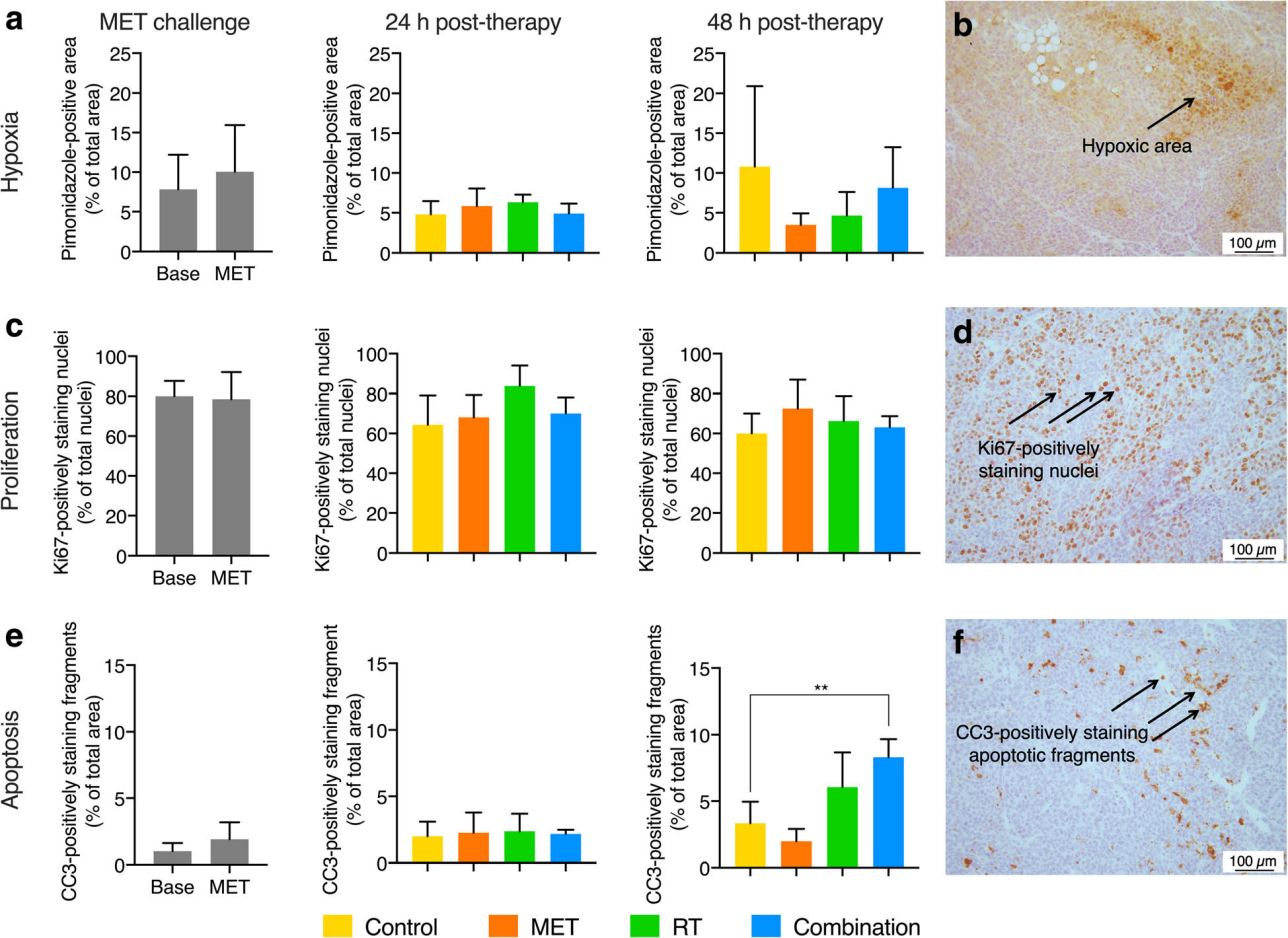
Histology satellite study. **a** No major differences in pimonidazole staining were observed between the different therapies at the different points in time. **b** Representative pimonidazole-stained tumor tissue of a metformin (MET)-treated animal. The arrow indicates a hypoxic area. **c** Same conclusions could be drawn for the Ki67 staining. **d** Representative Ki67-stained tumor tissue of a metformin-treated animal. Arrows indicate Ki67-positively staining nuclei. **e** 48 h post-therapy, the cleaved caspase-3 (CC3)-positive fraction was significantly increased in combination-treated tumors as compared to control. **f** Representative CC3-stained tumor tissue of a metformin-treated animal. Arrows indicate CC3-positively staining apoptotic fragments. **, *p *< 0.001; base, baseline; RT, radiotherapy

Figure 5 summarizes the results of the histology study. No immediate effect of metformin on tumor hypoxia could be detected with pimonidazole. Nevertheless, visually, we concluded that 48 h post-therapy hypoxia was decreased in metformin-treated animals as compared to controls (3.48% ± 0.83% vs. 10.79% ± 5.06%; *p* = 0.3). However, due to the large standard error in the control group, significance was not reached (Fig. 5a, b). No such differences were observed in the Ki67 staining (Fig. 5c, d). For CC3, compared to controls (3.33% ± 0.8%), the percentage of apoptosis was visually higher in the radiotherapy group (6.06% ± 1.30%; *p* = 0.1) and significantly higher in the combination group (8.31% ± 0.78%; *p* = 0.01) 48 h after therapy administration (Fig. 5e, f).

## Discussion

In Colo205 CRC xenograft tumors, baseline [^18^F]HX4 μPET acted as a prognostic biomarker for TDT. Moreover, acute metformin administration generated a decrease in [^18^F]HX4 TBR of approximately 9%. This finding confirms our earlier observations in A549 NSCLC xenograft tumors, in which a 10% reduction in [^18^F]HX4 TBR was measured [10]. However, metformin’s hypoxia reduction did not improve radiotherapy outcome in Colo205 tumors in the current study.

Several hypotheses may explain this unexpected lack of radiosensitizing effect of metformin, which opposes previous observations made in HCT116 and A549 xenograft tumors [8, 10]. First, we observed a significant increase in tumor volume in both the animals of the metformin and saline group in the 24 h time span between their baseline and follow-up scan, which we had not observed in our similar study in the NSCLC model (427 ± 36 mm^3^ to 418 ± 37 mm^3^; *p* = 0.3; unpublished data). This observation was confirmed in the high baseline Ki67 expression in the histology study. Both findings suggest that our experiment was initiated in a highly proliferative phase of tumor growth. Since tumors with a high proliferative activity have been shown to be more responsive to radiotherapy [18], the additive effect of metformin may have been masked by the already high susceptibility to the effects of radiotherapy of the tumors. Indeed, the applied dose of radiotherapy on itself was unexpectedly very effective with the median TDT almost being sevenfold higher in all radiotherapy-treated animals as opposed to controls (i.e., 41 days and 43 days vs. 6 days), without any additional effect of metformin. These observations were also reflected in the histology study, where 48 h after irradiation increased rates of apoptosis could be observed in both radiotherapy groups. This was however not seen in the Ki67 labeling index. Nevertheless, our results are in line with those obtained in head and neck cancer xenografts treated with a single dose of 10 or 20 Gy, where no changes in Ki67 expression were observed 24 h or 48 h after therapy [19]. On top of this, high proliferation may be coupled too quickly changing oxygenation dynamics [20]. Thus, the degree of hypoxia may have changed in the time span between the μPET scans and therapy administration, thereby potentially hampering metformin’s radiosensitizing capacities [5, 10, 21]. This assumption may be supported by the observation that, as opposed to its modulatory effects on [^18^F]HX4, metformin did not affect pimonidazole staining. However, this ex vivo technique only allows inter-animal assessment, as opposed to the intra-animal differences detected with μPET, and is prone to sampling error. Moreover, the limited sample size of the histology cohort (in order to limit the number of animals in this exploratory satellite study) provided lower statistical power.

In this regard, it could also be argued that the administered radiotherapy dose of 15 Gy was too high to see the radiosensitizing effect of metformin. Nevertheless, the 15 Gy dose was based on comparable research [8] in a CRC model with a radiosensitivity profile similar to Colo205 [22] and was, moreover, consciously administered as a single exposure, since it has been suggested that the reoxygenation that can occur during the course of fractionated radiation regimens may compromise the efficacy of radiosensitizers [23, 24], in this way potentially hampering the interpretation of their sensitizing capacities which we aimed to in the current study.

A second hypothesis for the lack of additive effect of metformin in Colo205, but not HCT116 and A549 tumors, is that metformin may also exert synergistic effects via antiproliferative mechanisms which are not related to hypoxia [25], for instance via altering DNA damage and repair kinetics [5, 6, 25]. In this regard, it should be taken into account that the A549 cell line is known to be particularly susceptible to such effects of metformin [26]. Conversely, the antiproliferative efficacy of metformin has been questioned in a variety of other models [27–34], including Colo205 cells in vitro [35]. Our current data also cast doubt on the metformin-sensitivity of Colo205 tumors in vivo since no effect of metformin on the Ki67 index could be detected. For Colo205, this observed lack of antiproliferative effect may be explained by the concept of BRAF^V600E^-driven resistance to metformin. Indeed, as opposed to HCT116 and A549 cells, the Colo205 cell line is BRAF^V600E^-mutated, and it has been shown in vivo that metformin had no effect on tumor growth [29] or even accelerated tumor growth [27] in BRAF^V600E^-mutated tumor models.

Importantly, however, irrespective of the lack of additive effect of metformin on radiotherapy efficacy, we showed that a higher tumoral baseline [^18^F]HX4 uptake was significantly associated with a poorer TDT, in this way implying that [^18^F]HX4 PET may have potential as a prognostic imaging biomarker. In a recent clinical pilot study in CRC patients, the applicability of PET with the prototype hypoxia tracer [^18^F]fluoromisonidazole ([^18^F]FMISO) was questioned, due to spill-in from rectal and bladder contents [36]. Importantly, [^18^F]HX4 is a third-generation 2-nitroimidazole compound that is relatively hydrophilic compared to the more lipophilic [^18^F]FMISO. Through this characteristic feature, faster clearance from normoxic tissue is achieved, resulting in better hypoxic-to-normoxic tissue ratios and thus better image contrast [14]. Similarly, the second-generation 2-nitroimidazole tracer [^18^F]fluoroazomycin arabinoside has already been suggested to be a valid alternative for hypoxia imaging in CRC tumors [37, 38]. Further research in orthotopic tumor models and patients is however warranted to confirm the superiority of [^18^F]HX4 over [^18^F]FMISO in CRC.

## Conclusions

In a Colo205 CRC xenograft model, we demonstrated that, despite its hypoxia-modulating effects, metformin was unable to sensitize tumors to the effects of radiotherapy. Additionally, we showed that baseline [^18^F]HX4 PET holds promise as a prognostic imaging biomarker in CRC.

## Data Availability

The datasets generated and/or analyzed during the current study are available from the corresponding author upon reasonable request.

## Abbreviations

[^18^F]FMISO: [^18^F]fluoromisonidazole
[^18^F]HX4: [^18^F]flortanidazole
μPET: Small-animal positron emission tomography
AMPK: Adenosine monophosphate-activated protein kinase
CC3: Cleaved caspase-3
CI: Confidence interval
CRC: Colorectal cancer
CT: Computed tomography
ETC: Electron transport chain
FITC: Fluorescein isothiocyanate
HR: Hazard ratio
HRP: Horseradish peroxidase
mTOR: Mammalian target of rapamycin
NSCLC: Non-small cell lung cancer
PET: Positron emission tomography
SEM: Standard error of the mean
TBR: Tumor-to-background ratio
TDT: Tumor doubling time
